# Determining the Severity of Atopic Dermatitis in Children Presenting in General Practice: An Easy and Fast Method

**DOI:** 10.1155/2009/357046

**Published:** 2009-11-18

**Authors:** Marjolein G. Willemsen, Rosalinda W. C. van Valburg, Pauline C. Dirven-Meijer, Arnold P. Oranje, Johannes C. van der Wouden, Heleen Moed

**Affiliations:** ^1^Department of General Practice, Erasmus MC—University Medical Center, P.O. Box 2040, 3000 CA Rotterdam, The Netherlands; ^2^General Practice, Dorpsstraat 61, 3927 BB Renswoude, The Netherlands; ^3^Department of Dermatology and Venereology (Pediatric Dermatology), Erasmus MC—University Medical Center Rotterdam/Sophia Children's Hospital, P.O. Box 2040, 3000 CA Rotterdam, The Netherlands

## Abstract

Assessment of the severity of atopic dermatitis (AD) is necessary to evaluate the disease process. This study evaluates and validates the TIS in children with AD presenting in general practice. Independent investigators determined the severity of AD using the TIS and the objective SCORAD. The interobserver agreement for the TIS and SCORAD was calculated, as was the correlation between TIS and SCORAD. The mean time to assess the TIS was less than one minute. A moderate-to-good agreement between the observers was found for the TIS (*κ* = 0.604 or 0.464), or SCORAD (*κ* = 0.695 or 0.700). There was an excellent correlation between TIS and SCORAD (*r*_*s*_ = 0.755–0.839). In conclusion, the TIS is an easy and fast method to score AD. Because of the moderate to good interobserver agreement and the high correlation with the SCORAD, we recommend the TIS to determine the severity of AD in general practice.

## 1. Introduction

It is important to determine the severity of atopic dermatitis (AD) when assessing the course of the disease, for example, before and after treatment [1]. Because there are no serologic markers that accurately reflect AD, measurements and severity are primarily based on signs and symptoms [2]. Valid and reliable clinical outcome measures are prerequisites for evidence-based practice [3].

Investigators can currently select from 20 different measurements of AD disease severity. Of these, only the EASI (Eczema Area Severity Index) [4], POEM (patient-oriented eczema measure) [5], and SCORAD (SCORing Atopic Dermatitis) index [6, 7] have been sufficiently validated to recommend their use in clinical trials and everyday practice [8].

The most widely used clinical scoring system is the SCORAD index [7]. The SCORAD index (including six objective items and two subjective issues) or the objective SCORAD (SCORAD from now on, without subjective measures) is an excellent system for clinical trials but is too complicated and time consuming (scoring takes about 7–10 minutes per patient) for a routine clinical setting. For this reason, the Three Item Severity score (TIS), a simplified version of the SCORAD, was developed [1, 9].

The TIS has been tested in a dermatology outpatient department; a high correlation and a moderate-to-fair interobserver agreement were found between the TIS and the SCORAD [9]. The Dutch College of General Practitioners' guideline on AD recommends treating patients according to the severity of AD [11]. The guideline acknowledges that the value of the TIS has not yet been established in general practice and recommends more research in this area [11].

Therefore, the present study explored whether the TIS is useful in children treated in general practice. For this, we evaluated and validated the TIS, and calculated the interobserver agreement for and the correlation between the TIS and the SCORAD.

## 2. Materials and Methods

### 2.1. Patient Selection

This prospective study was conducted in general practices in greater Rotterdam and a regional Child Health Center (Renswoude area) between November 2007 and March 2008. Included were all patients with AD under 7 years of age. The children were selected from the computerized files of general practitioners (GPs). Children were selected either by diagnosis, coded according to the International Classification of Primary Care (ICPC) [12], or by prescribed medication, coded according to the Anatomical Therapeutic Chemical (ATC) classification scheme [13].

Patients were selected using the ICPC code S87 (Atopic Dermatitis), and/or ATC codes for topical treatment of AD (zinc products, soft paraffin, and fat products, other emollients and protectives, tars, topical corticosteroids, all different potencies), and according to the inclusion and exclusion criteria (Table 1). 

The study was approved by the ethics committee of Erasmus MC. All parents provided written informed consents to participate in the study.

### 2.2. Materials

The SCORAD index consists of the interpretation of the extent of the disorder, that is, the intensity, composed of six items (erythema, oedema/papules, effect of scratching, oozing/crust formation, lichenification, and dryness), and two subjective symptoms (itch and sleeplessness) [1]; the maximum score is 103 points (Table 2).

In the SCORAD the subjective symptoms (itch and sleeplessness) are not assessed; the total score is 83 points (Table 2) [6]. “Bonus points” for disfiguring lesions or functional limiting lesions will receive 10 extra points (Table 2). 

The TIS is the sum of the three items: erythema, oedema, and excoriations (scored on a scale from 0 to 3); each item should be scored on the most representative lesion, that is, a lesion which represents the mean intensity for that item (Table 2) [9]. The TIS items are identical to their SCORAD counterparts.

Two investigators (MW and RVV) were trained by a pediatric dermatologist (APO) to complete the TIS and SCORAD. To assess interobserver variability, both investigators determined the severity of AD in all children simultaneously using the TIS and SCORAD. As a third observer, a GP (PDM) simultaneously scored a subset of patients. 

During the examination, the observers scored the severity of AD using first the TIS and then using the SCORAD. 

To assess usability of the TIS the observers recorded the time required to assess the total TIS per patient. 

All patients were observed at 3- to 4-week intervals, and digital images of the most representative lesions were made in all patients.

Because the symptoms of AD can fluctuate greatly, all the observers examined the patients during the same visit; during each examination, the other observers were in a separate room. The results of each examination were immediately stored in a sealed envelope, ensuring that the other observers were unaware of these results. The envelopes remained sealed until the last observer had examined the patients for the second time.

### 2.3. Statistical Analysis

The total scores of the TIS and SCORAD were calculated per patient for each observer. The total scores of the TIS (0–9) and the SCORAD (0–83/93) were then categorized into three groups: mild, moderate, and severe. We chose to categorize the scores into these groups since treatment of AD is based on this classification. The scores were categorized as shown in Table 2.

Statistical analysis was performed with SPSS version 15 (SPSS Inc, Chicago) and STATA version 10 (StataCorp, College Station). Interobserver agreement for both the SCORAD and TIS (categorized scores) was calculated using Cohen's kappa. Agreement for each item separately (lichenification, oozing/crusts, dryness, erythema, oedema/populations, and excoriations) was also calculated using Cohen's kappa. We consider an agreement between the observers above 0.75 as an excellent agreement and below 0.40 as a poor agreement; results between 0.4 and 0.75 were regarded as fair to good [14]. Agreement regarding the extent of the disease was calculated with the intraclass correlation coefficient (ICC).

The correlation between the TIS and SCORAD (uncategorized scores) was calculated using Spearman's rank correlation. The correlation between the categorized SCORAD (i.e., classification into mild, moderate, and severe) and categorized TIS was calculated using the ICC.

The correlations between SCORAD (the uncategorized scores) and the different items (lichenification, oozing/crusts, dryness, erythema, oedema/papulations, excoriations, and the extent) and between the TIS (the uncategorized scores) and the three items (erythema, oedema/populations, and excoriations) were calculated using Spearman's rank correlation.

## 3. Results

### 3.1. Study Population

A total of 45 GPs agreed to participate in the study. A total of 278 patients with an age below 7 years and with a history of AD (ICPC S87) or use of medical treatment for AD were selected in the database of the GP. These selected patients were invited by mail to participate. Of these, 89 had self-reported complaints of AD at the moment and were willing to participate. Finally 66 patients were included in the study. Twenty three children were excluded for the following reasons: 12 had no AD complaints at the time of inclusion, 3 parents were unable to fill in the consent forms and another 8 were excluded because their answer forms were received after the inclusion period had closed. Thus, the final study population consisted of 66 patients (57.6% girls, mean age of 31 months—range 0.5–83 months). Of these, 50 were examined by two observers and 16 by three observers. All 66 patients were available for the first visit, and 65 patients were observed during the second visit.

### 3.2. Severity of AD

The mean TIS scores were 2.1 (SD = 1.1, determined at the first visit) and 1.8 (SD = 1.0, at the second visit). The mean SCORAD scores were 13.5 (SD = 8.7) at the first visit and 11.9 (SD = 7.8) at the second visit. The severity of AD was predominantly mild, accounting for 73% of the cases according to the TIS (score 0–2) and for 70% of the cases according to the SCORAD (0–14). Moderate AD accounted for 24.6% according to the TIS (score 3–5) and for 27.6% according to the SCORAD (score 15–40). Severe AD accounted for 2.4% in both scoring systems.

### 3.3. Evaluation of the TIS and SCORAD

The mean time to assess the TIS per patient was 43 seconds (range 7*–*170). Moderate-to-good interobserver agreement (weighted *κ* = 0.604 for visit 1 and 0.464 for visit 2) was found between the two observers for the categorized TIS (i.e., severity classified into mild, moderate, and severe). Similar results were found in a smaller group where patients were observed by three observers (Table 3). 

For the SCORAD (categorized into mild, moderate, and severe) the interobserver agreement also showed a good agreement (*κ* = 0.665 for first visit and *κ* = 0.776 for second visit).

### 3.4. Correlation between the TIS and SCORAD

**Figure 1:**
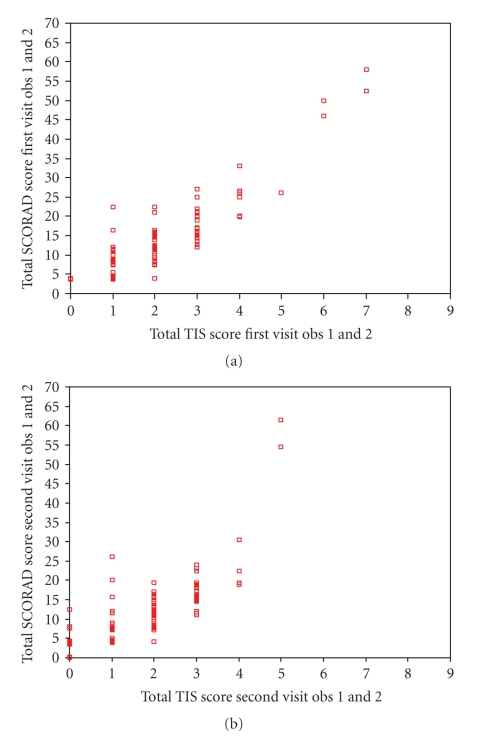
Correlation between TIS and SCORAD. Observations from the two independent observers (obs 1 and 2) are displayed in one figure ((a) first visit, (b) second visit).

We calculated the correlation between the TIS and SCORAD. For all three observers for both visits, a good correlation was found between the two scoring methods (Table 4, Figures 1(a) and 1(b)). The correlation between the categorized TIS (i.e., severity classified into mild, moderate, and severe, Table 2) and categorized SCORAD (same classification) was also good or excellent for all observers at both visits (*r*_*s*_ = 0.637 to 0.985; *P* = .01; ICCs 0.622 to 0.933). 

### 3.5. Correlation between Separate Intensity Items and TIS or SCORAD

The correlation between the TIS and the separate intensity items (erythema, oedema, and excoriations) was calculated. For all observers, all intensity items correlated well with the TIS (*r*_*s*_ 0.611–0.980 for erythema; 0.604–0.838 for oedema; 0.639–0.860 for excoriations). The items erythema, oedema, and excoriations also correlated well with the SCORAD for all observers (*r*_*s*_ from 0.495 to 0.826). Regarding the other items of the SCORAD, the items dryness and extent showed a moderate-to-good correlation with the SCORAD for all observers (*r*_*s*_ 0.324 to 0.855 for dryness and 0.697 to 0.963 for extent). Only the items oozing/crusts and lichenification showed a weak correlation with the SCORAD (*r*_*s*_ from 0.197 to 0.638).

### 3.6. Interobserver Agreement on Separate Intensity Items

The interobserver agreement for the separate intensity items showed highly variable kappa values. Only the item “erythema” showed a fair-to-good agreement between the observers (*κ* = 0.603 or 0.621). Also a good agreement for the item “extent” (item A of the SCORAD) was found between the observers (ICC 0.761 and 0.857, for visit 1 and 2, resp.).

## 4. Discussion

In the present study a moderate-to-good interobserver agreement was found between the observers for the TIS. The overall agreement between observers was sufficiently good to recommend use of the TIS to assess the severity of AD in general practice. The TIS is a simple method to determine the severity of AD, and assessment of the total TIS took about 43 seconds per patient. Therefore, the TIS is suitable for use in daily practice. Because a good correlation was found between the TIS and the different intensity items, we conclude that these items (erythema, oedema, and excoriations) are suitable determinants to evaluate the severity of AD with the TIS. 

In accordance with other studies [1, 9], we found a good correlation between the TIS and SCORAD. The classification of the severity of AD into mild, moderate, and severe also showed good correlations, but were lower than the uncategorized scores. A possible explanation is that these categorized scores were rearranged on an arbitrary line. Thereby, some patients had a difference of only one point between the two observers on the TIS, but were placed in a different category (mild, moderate, or severe). When the patient scores were uncategorized, these minor differences between the observers had no influence on the calculated correlation; therefore, these correlation scores are higher. The high correlation between TIS and SCORAD is not surprising, because the TIS is a simplified version of the SCORAD. 

For the SCORAD, a good interobserver agreement was found between all observers. Similar to clinical trials in which good interobserver agreement for the SCORAD has been established [1, 9], the SCORAD also showed good agreement in our general practice patients. We conclude that, although SCORAD is an effective scoring system and is suitable to determine the severity of AD in clinical trials, it is too extensive and time consuming for use in daily care in general practice.

Because the items erythema, oedema, and excoriations correlated well with the SCORAD, we conclude that implementing these items in the TIS is a feasible choice. 

The interobserver agreement between the different intensity items showed a wide range of differences, therefore these items alone cannot be used to determine the severity of AD. 

In contrast to previous studies [6, 15], the item “extent” (of the SCORAD) showed good agreement between all our observers. Furthermore, we found a good correlation between the SCORAD and the item “extent.” An explanation for this good correlation could be the fact that children who visit a GP for their eczema in general have a mild form of eczema. The extent of this mild form is easier to score, since it is all in the same (low) range. The extent of AD is not assessed in the TIS; its addition could provide additional information about the severity of AD in children who visit the GP for eczema.

### 4.1. Limitations of the Present Study

A weakness of this study is that, because we included patients who had already received treatment for AD, the severity of their AD was probably diminished at the time of the first visit (several days/weeks after the visit to the GP). Nevertheless, when comparing AD complaints assessed during the first and second visit, a decrease in these complaints was observed at the second visit. This indicates that AD complaints during the first visit were determined during a period with fair complaints of AD.

Because of the mild distribution of the severity of AD in our study population, it is difficult to predict whether our results (e.g., interobserver agreements and correlations) are comparable with results in other populations. The interobserver agreement between the three observers was only available for a relatively small sample; therefore, these results should be considered with caution. The interobserver agreement between the two observers could be calculated for a much larger sample size.

### 4.2. Conclusion

In conclusion, the TIS is an easy and fast scoring system. Because of the moderate-to-good interobserver agreement and the high correlation with the SCORAD, we recommend that the TIS be used to determine the severity of AD in general practice.

## Figures and Tables

**Table 1:** Inclusion and exclusion criteria.

	Inclusion criteria	Exclusion criteria
	– Aged 0–6 years	– Chronic disease other than AD, asthma, food intolerance, or allergic rhinitis
Patient selection (computer search)	– Diagnosis of AD by GP	– Psychological problems which could influence follow-up
	– Had consultation for AD complaints within last 3 months or repeated their prescriptions for AD treatment in last 3 months	– GP checked the selected patients and could exclude patients

Telephone questionnaire	– AD complaints at time of telephone questionnaire (excoriations and skin lesions)	– Short-term illness at time of telephone questionnaire
		– Parent or caretaker unable to read or write Dutch or English

First visit	– Informed consent	– Other skin conditions precluding proper assessment of AD severity
	– Diagnosis of AD using Williams' criteria [10]	– Short-term illness

AD: atopic dermatitis, GP: general practitioner.

**Table 2:** Classification of the severity of atopic dermatitis for the SCORAD-index, the (objective) SCORAD, and Three Item Severity score (TIS)^±^.

Instrument	Classification of severity of AD		
	Mild	Moderate	Severe
SCORAD-index	0–24	25–50	51–103
(Objective) SCORAD	0–14	15–40	41–83/93
TIS	0–2	3–5	6–9

^±^Adapted from Oranje et al. [1].

**Table 3:** Interobserver agreement for the TIS (categorized into mild, moderate, or severe).

	First visit (*n* = 66)	Second visit (*n* = 65)
Observer 1 versus 2	*κ* = 0.562	*κ* = 0.464
	Weighted *κ* = 0.604	Weighted *κ* = 0.464

	First visit (*n* = 16)	Second visit (*n* = 15)

3 observers	*κ* = 0.758	*κ* = 0.481

**Table 4:** Correlation between TIS and SCORAD.

	First visit (*n* = 66)	Second visit (*n* = 65)
Observer 1; TIS versus SCORAD	*r*_*s*_ = 0.807 (*P* = .01)	*r*_*s*_ = 0.755 (*P* = .01)
Observer 2; TIS versus SCORAD	*r*_*s*_ = 0.791 (*P* = .01)	*r*_*s*_ = 0.839 (*P* = .01)

	First visit (*n* = 16)	Second visit (*n* = 15)

Observer 3; TIS versus SCORAD	*r*_*s*_ = 0.809 (*P* = .01)	*r*_*s*_ = 0.791 (*P* = .01)

*r*_*s*_, Spearman's rank.
